# Cognitive Function and Postural Control Strategies in Relation to Disease Progression in Patients with Parkinson’s Disease

**DOI:** 10.3390/ijerph191912694

**Published:** 2022-10-04

**Authors:** Monika Zawadka-Kunikowska, Jacek J. Klawe, Małgorzata Tafil-Klawe, Monika Bejtka, Łukasz Rzepiński, Mirosława Cieślicka

**Affiliations:** 1 Department of Human Physiology, Ludwik Rydygier Collegium Medicum in Bydgoszcz, Nicolaus Copernicus University, Karłowicza 24, 85-092 Bydgoszcz, Poland; 2 Department of Hygiene, Epidemiology, Ergonomy and Postgraduate Education, Ludwik Rydygier Collegium Medicum in Bydgoszcz, Nicolaus Copernicus University in Torun, M. Sklodowskiej-Curie 9, 85-094 Bydgoszcz, Poland; 3 Department of Neurology, 10th Military Research Hospital and Polyclinic, 85-681 Bydgoszcz, Poland; 4 Sanitas-Neurology Outpatient Clinic, Dworcowa 110, 85-010 Bydgoszcz, Poland

**Keywords:** Parkinson’s disease, dual task, cognitive function, executive functions, postural control strategies

## Abstract

Aim: This study assessed the influence of performing an additional cognitive task on center of pressure (COP) displacement in the early and advanced stages of patients with Parkinson’s disease (PD) compared to age-matched healthy controls (HCs). Methods: The study included 40 HCs and 62 patients with PD: early PD (*n* = 38) and advanced PD (*n* = 24). COP parameters were determined by static posturography during quiet standing with open eyes (ST, single task) and simultaneous performance of a cognitive task (DT, dual task). Cognitive functioning was examined with a Mini Mental State Examination, number-counting-backward test, and number of enunciated words during DT. Results: In the advanced-PD group, DT significantly reduced the sway radius (*p* = 0.009), area of stabilogram (*p* = 0.034), medio-lateral length (*p* = 0.027), and velocity (*p* = 0.033) compared to ST. In HCs, DT showed a significant increase in the sway radius (*p* = 0.006), total length (*p* = 0.039), sway velocity (*p* = 0.037), anterior–posterior length, and sway velocity. Both PD groups showed worse cognitive performance compared to HCs. Conclusions: Both early and advanced patients with PD showed significant delay in cognitive performance associated with executive function compared to the HCs. During additional cognitive tasks, patients with advanced stages of PD may reduce stabilographic parameters in medio-lateral direction, and this is probably an adaptive strategy to restore balance.

## 1. Introduction

Postural instability, together with orthostatic hypotony and freezing, are the most common causes of fall-related injuries in moderate-to-advanced stages of Parkinson’s disease (PD) [1,2,3]. Many factors have been implicated as etiologies of postural impairment in patients with PD. Failure in motor programming due to reduced activity in the motor cortical areas, impairment of cognitive functions, and disturbances in posture–gait areas in the brainstem and the dopaminergic and cholinergic systems have been suggested as the likely mechanisms [4,5,6]. Postural control is not a completely automatic process, but it requires visual, somatosensory, and vestibular inputs, in addition to attentional resources [7,8].

Studies using dual-task paradigms suggest that the simultaneous performance of two component tasks is especially challenging, since executive function and ability to divide attention is often impaired even in early stages of PD [9,10]. Postural control during a dual task is also associated with increased activity in the central motor networks, including the prefrontal and motor cortex, as this has been reported in the elderly and in patients with PD [11]. Additionally, these brain regions are the most sensitive to age-related effects [12]. On the other hand, it has been suggested that increased activation of the prefrontal and motor cortex during cognitive load may be related to altered sensorimotor functioning reducing the sensitivity of the peripheral reflexes which underlie gait instability in patients with PD [13].

Numerous studies have documented that additional cognitive tasks can interfere with motor performance in both healthy elderly individuals and patients with PD [4,6,13,14,15,16]. Some researchers suggest that dual tasking does not negatively influence balance during performance when competing tasks where cognitive resources are available. Hence, with more complex cognitive tasks when available resources are depleted, favorable performance on one task may require shifting of resources away from other tasks, depending upon their priority [6,8,17,18].

Previous studies suggest that healthy individuals, including young and older adults, may spontaneously prioritize posture control over cognitive task performance, suggesting the use of a “posture first strategy”. In contrast, patients with PD inappropriately use a posture-second strategy, focusing attention on a secondary task [14,19,20]. However, recent reports do not provide consistent evidence to explain the influence of dual-task performance on postural sway in PD. Some investigators report that PD patients during execution of cognitive tasks have larger amplitudes of postural sway compared to age-matched controls [21,22,23]. However, others have found smaller postural sway in patients with PD [15] or no difference between the groups at all [24]. To our knowledge, many studies have analyzed the postural-sway characteristics of patients with PD compared with healthy controls during dual tasking [15,21,23,24,25]; nevertheless, the changes of the stabilographic parameters and cognitive function in early and advanced stages of PD have not been very well studied. Moreover, patients with PD in stage four of the H–Y scale are often excluded from studies.

In general, transition from Hoehn–Yahr (H–Y) stages two to three is considered a milestone in PD, suggesting significant disability, with loss of independent function [26,27].

A better understanding of postural control mechanisms and attentional processing is essential for developing effective rehabilitation interventions for improving functional mobility in different stages of PD associated with disease progression and accompanying age-related neurodegenerative changes [28].

The main aim of this study was to analyze the influence of performing an additional cognitive task on COP displacement in early and advanced stages of patients with Parkinson’s disease compared to age-matched heathy elderly subjects. We hypothesized that the PD groups would present poorer dual-task performance due to the changes in physical and cognitive functions, in particular in the executive functions during the disease progression. The second aim was to investigate the relationship between postural instability and age and executive function (in particular, attention and working memory).

## 2. Methods

### 2.1. Research Design and Study Sample

**Figure 1:**
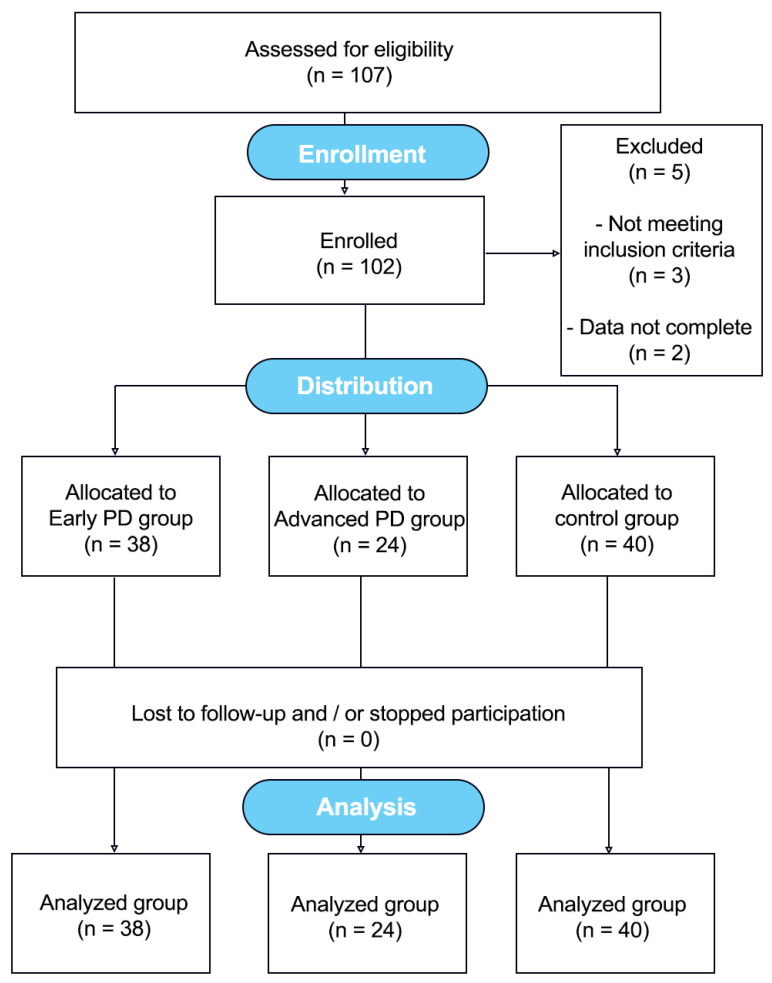
Flow diagram showing the course of the study [29].

A total of 102 individuals were examined, including 40 healthy subjects without neurological impairment and 62 patients with confirmed idiopathic PD. The data collection was conducted from 2012 to 2018. Figure 1. The clinical characteristics of the sample are displayed in Table 1. Patients were recruited from the Jan Biziel University Hospital No. 2 in Bydgoszcz and the local PD associations. The controls were interested subjects from the local community (Northern Poland).

The diagnosis of PD was based on the assessment of a neurologist, according to the criteria of the UK Parkinson’s Disease Society Brain Bank. The clinical stage of PD was assessed by using the H–Y scale [30]. The H–Y scale ranges from 1 (mild Parkinsonian symptoms) to 5 (severe disability and complete dependence on others). Patients with PD were allocated to two groups according to the clinical stage of the H–Y rating scale: early PD (stages 1 and 2) and advanced PD (stages 3 and 4). The group of early PD comprised 38 individuals, aged 46–82 years, disease duration 1–10 years, H–Y stage (mean 1.68 ± 0.47). The group of advanced PD comprised 24 individuals, aged 60–81 years, disease duration 1–25 years, H–Y stage (mean 3.25 ± 0.44). Each patient with PD was examined approximately 2 h after intake of his/her regular anti-parkinsonian medication. The levodopa equivalent daily dose (LEDD) was computed for each participant according to the formula by Tomlinson et al. [31].

All testing procedures took place while patients were on medication. The control group consisted of 40 healthy subjects, aged 52–89 years. The inclusion criteria in the PD group were as follows: confirmed diagnosis of idiopathic PD, H–Y stage between 1 and 4, ability to maintain standing position for at least 96 s, logical verbal contact with the subject, and lack of musculoskeletal pathology that could impair balance during quiet standing. The exclusion criteria for PD were as follows: severe dyskinesia and/or motor fluctuation, action, or postural tremors. The inclusion criteria for healthy individuals included the lack of neurological and orthopedic disorders, logical verbal contact with the subject, and other diseases and/or conditions that could potentially impair balance. The subjects did not use psychotropic medications or show signs of depression (Geriatric Depression Scale score < 10). The criteria were in accordance with a previous study [32].

The research protocol was approved by the Bioethical Committee of Collegium Medicum in Bydgoszcz, Nicolaus Copernicus University in Torun (protocol no. KB 405/2009). All the subjects voluntarily signed informed consent forms before the study procedure.

### 2.2. Assessment of Postural Stability

Center-of-pressure (COP) displacements were registered by using a diagnostic system comprising a posturographic force plate (Pro-Med version 2010, Janusz Olton, Legionowo, Poland) and computer software. The subjects were instructed to stand naturally on the static force plate (400 × 400 × 55 mm), with four pressure sensors (one in each corner) measuring the forces exerted by the subjects on the support surface. All tests were performed with the toes 30° apart and the distance of 2 cm between the subject’s heels. All subjects were asked to avoid strenuous exercise 24 h prior to the assessment.

Postural control was assessed in two experimental conditions: quiet standing with open eyes (single task—ST) and simultaneous performance of cognitive task of counting backward by one digit from 50 during standing (dual task—DT). Each condition consisted of two 32-s trials with 2 min of rest in between to eliminate potential discomfort associated with standing still for an extended period of time. All the functions have been successfully used in recent clinical studies [32,33].

The following parameters were analyzed: mean radius (R) (mm), standard deviation of sway radius (SD R), stabilogram’s area (P) (mm), mean total length (L) covered by the COP (mm), mean sway velocity (V) (mm/s), standard deviation of sway velocity (SD V), anterior–posterior sway velocity (V_A-P)_ (mm/s), standard deviation of anterior–posterior sway velocity (SD V_A-P_), medio-lateral sway velocity (V_M-L_) (mm/s), standard deviation of anterior–posterior medio-lateral sway velocity (SD V_M-L_), anterior–posterior length of stabilogram (L_A-P_) (mm), and medio-lateral length of stabilogram (L_M-L)_ (mm).

### 2.3. Assessment of Cognitive Function

The Mini Mental State Examination (MMSE) was applied to obtain the overall level of cognitive functioning. The final score of this test includes points for orientation in place and time, recall of earlier named prompts after a short period of time, repeating three named prompts, attention and calculation, naming two items, performing verbal commands, writing, and copying two overlapping pentagrams [34] Attention and executive function were examined by using the counting-backward test (CBT) in baseline conditions. CBT was used to evaluate working memory, as well as the ability to focus and sustain attention. [35] The subjects were instructed to count back as quickly as possible, beginning from 20 to 0. A longer time interval required to complete the test signifies attention and executive-function deficits [33]. During DT, subjects were asked to count backward by one digit, from 50, while standing. The mean number of enunciated words were calculated (counting backward by one digit, from 50, while standing).

### 2.4. Statistical Analysis

All data are presented as mean ± SD. Normal distribution of the study variables was verified with the Shapiro–Wilk test. The statistical significance of differences between parameters in the two groups was verified with the Student’s *t*-test or Mann–Whitney U test. Cognitive function was compared between groups. A least-significant-difference (LSD) post hoc test was used when a significant difference was found in the one-factor ANOVA. To investigate the dual-task effects in postural sway, we used a two-way (3 groups × 2 tasks) repeated measures analysis of variance (ANOVA) between groups (early PD, advanced PD, and controls) and within task (single task/dual task). Partial eta-squared (ŋ_p_^2^) was used to calculate the effect sizes of the statistical results. Norms for interpreting η^2^ are 0.01  = small effect, 0.06  = moderate effect, and 0.14  = large effect [36]. Bonferroni’s test was used in the case of significant differences. The strength and significance of correlation between the selected variables were calculated by using the nonparametric Spearman’s test. The covariance model (ANCOVA) was used to assess the effect of age on COP parameters and cognitive functions. The adjusted R-squared was calculated. The level of significance for all tests was set at *p* < 0.05. All calculations were conducted with STATISTICA 13.0 PL statistical package (StatSoft, Kraków, Poland.

## 3. Results

### 3.1. Subjects Characteristics

No differences were observed between the early PD, advanced-PD, and control groups in age (*p* = 0.3). There were significant differences between the early PD and advanced-PD groups in terms of disease duration, H–Y stage, and levodopa equivalent daily dose (LEDD) (Table 1). Rehabilitation treatment (i.e., physical exercise) was used by 16 (43.2%) subjects from the early PD group and 13 (54.2%) from the advanced-PD group. All the healthy subjects were active individuals.

### 3.2. Assessment of Cognitive Function

Overall, there were significant differences between the PD patients and controls in terms of all cognitive tests (Table 1). The MMSE was significantly different between groups: F (2.98) = 4.64, *p* = 0.018). The one-way ANOVA revealed that the control group had significantly higher MMSE scores compared to the early PD group (*p* = 0.034) and advanced-PD group (*p* = 0.005).

The CBT differed significantly between the groups: F (2.98) = 7.75, *p* < 0.001). The early PD (*p* = 0.001) and advanced-PD (*p* = 0.003) groups had a significantly longer mean time duration of the test compared to the control group. Moreover, the number of words enunciated in the DT condition was significantly different between groups: F(2.98) = 5.48, *p* = 0.006. The control group had a significantly higher mean number of words compared to the early PD group (*p* = 0.004) and advanced-PD group (*p* = 0.012).

### 3.3. Assessment of Postural Stability (COP Parameters)

**Figure 2:**
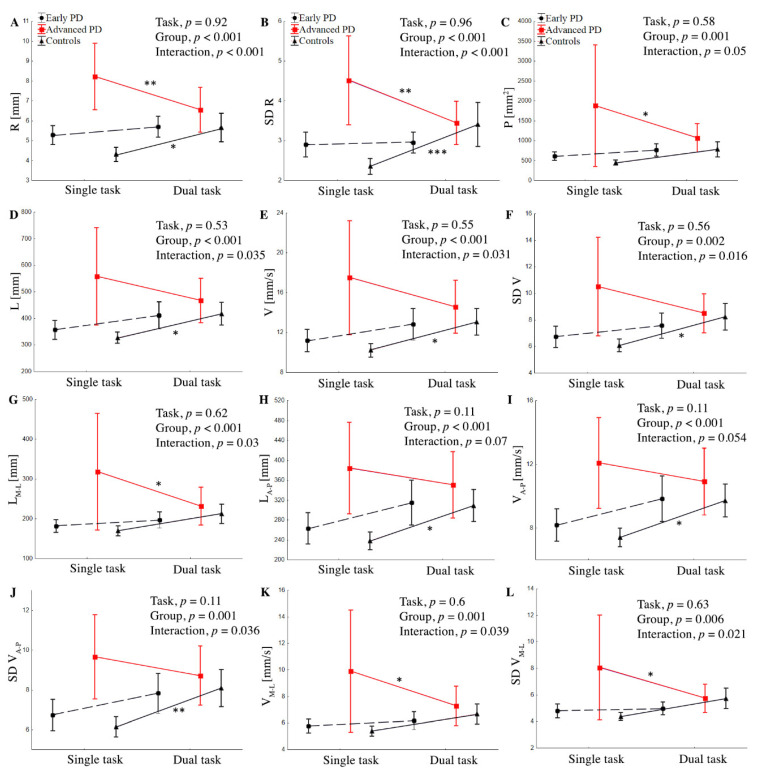
Group mean values (±SD) of R (**A**), SD R (**B**), P (**C**), L (**D**), V (**E**), SD V (**F**), L_M-L_ (**G**), L_A-P_ (**H**), V_A-P_, (**I**), SD V (**J**), V_M-L_ (**K**) SD V_M-L_ (**L**) during single and dual task and interaction between group and task for stabilographic parameters. ANOVA results for means’ difference between the control and PD groups. Statistically significant differences are indicated as * *p* < 0.05, ** *p* < 0.01, and *** *p* < 0.001.

The two-way ANOVA revealed a difference between groups (F (24,370) = 0.74, *p* < 0.001), but not for task (F (12,185) = 0.94, *p* = 0.5), thus indicating that the influence of performing an additional task on COP displacement was similar for both the PD and control groups. There was a significant interaction between group and task (F (24,370) = 0.81, *p* = 0.029). All groups’ task main effects and interactions are presented in Figure 2. No significant differences in any stabilographic parameters were observed between the control and early PD groups in all two conditions (*p* > 0.05).

#### 3.3.1. Mean Sway Radius (R), Standard Deviations of Mean Radius (SD R), and Area of Stabilogram (P)

The two-way ANOVA revealed a difference between groups for R (F (24,372) = 18.9, *p* < 0.001, ŋ_p_^2^ = 0.161), SD R (F (24,372) = 10.7, *p* < 0.001, ŋ_p_^2^ = 0.099), and P (F (24,372) = 7.1, *p* < 0.0001, ŋ_p_^2^ = 0.068), but not for task. There was a significant interaction between group and task for R (*p* < 0.001, ŋ_p_^2^ = 0.068) and SD R (*p* < 0.001, ŋ_p_^2^ = 0.082), indicating that the effects of the task were different in the three groups (Figure 2).

The post hoc showed that, during the ST, the subjects from the advanced-PD group showed significantly higher values of R, SD R, and P compared to the early PD and control groups, *p* < 0.001. In the advanced-PD group, DT significantly reduced R (*p* = 0.009), SD R (*p* = 0.009), and P (*p* = 0.034), whereas the control group showed a significant increase in R (*p* = 0.006) and SD R (*p* = 0.009) compared to the ST. In the early PD group, we did not find a significant difference between single and dual tasks; however, variables tended to be higher in the DT condition. Similarly, during the DT, subjects from the advanced-PD group showed significantly higher R, SD R, and *p*-values compared to the early PD and control groups (Figure 2), but without statistical significance, *p* > 0.05.

#### 3.3.2. Sway Velocity, Standard Deviation of Sway Velocity, Medio-Lateral and Anterior—Posterior Sway Velocity, and Standard Deviations of Medio-Lateral and Anterior—Posterior Sway Velocity

**Figure 3:**
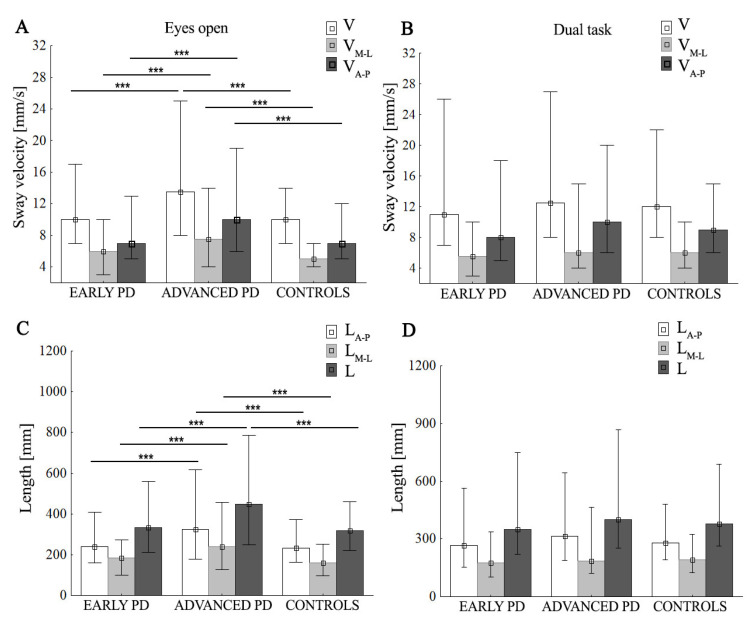
Group mean values (±SD) of sway velocity (V), mean sway velocity in medio-lateral (V_M-L_) and anterior–posterior (V_A-P_) (**A**,**B**), total length of stabilogram (L), medio-lateral length of stabilogram (L_M-L_), and anterior–posterior (L_A-P_) length of stabilogram (**C**,**D**), during eyes-open/dual task. Statistically significant differences are indicated with *** *p* < 0.001.

The two-way ANOVA revealed a difference between groups for V (F (24,372) = 9.00, *p* < 0.001, ŋ_p_^2^ = 0.084), SD V (F (24,372) = 6.58, *p* = 0.002, ŋ_p_^2^ = 0.063), V_M-L_ (F (24,372) = 6.89, *p* = 0.001, ŋ_p_^2^ = 0.065), V_A-P_ (F (24,372) = 8.6, *p* < 0.001, ŋ_p_^2^ = 0.082), SD V_M-L_ (F (24,372) = 5.28, *p* = 0.006, ŋ_p_^2^ = 0.051), and SD V_A-P_ (F (24,372) = 7.85, *p* = 0.001, ŋ_p_^2^ = 0.074). There was significant interaction between the group and task for V (*p* = 0.031, ŋ_p_^2^ = 0.035), SD V (*p* = 0.016, ŋ_p_^2^ = 0.041), V_M-L_ (*p* = 0.039, ŋ_p_^2^ = 0.039), SD V_M-L_ (*p* = 0.021, ŋ_p_^2^ = 0.038), and SD V_A-P_ (*p* = 0.036, ŋ_p_^2^ = 0.033), indicating that the effects of the task were different in the three groups (Figure 2). The post hoc showed that, in the advanced-PD group, the dual task significantly reduced V_M-L_ (*p* = 0.033) and SD V_M-L_ (*p* = 0.027), whereas the control group showed a significant increase in V (*p* = 0.037), SD V (*p* = 0.02), and SD V_A-P_ (*p* < 0.001). No significant differences between ST and DT were observed in the case of advanced PD subjects for V, SD V, V_A-P_, and SD V_A-P_, and in controls for V_M-L_ and SD V_M-L_ (*p* > 0.05). In the early PD group, we did not find a significant difference between ST and DT, although V, SD V, V_A-P_, SD V_A-P_, V_M-L_, and SD V_M-L_ tended to be higher in the DT test. During the DT, subjects from the advanced-PD group showed significantly higher V, SD V, V_A-P_, SD V_A-P,_ V_M-L_, and SD V_M-L_ values compared to the early PD and control groups (Figure 3A,B), but without statistical significance (*p* > 0.05).

#### 3.3.3. Total Length (L) and Medio-Lateral and Anterior—Posterior Length of Stabilogram (L_M-L_ and L_A-P_, respectively)

The two-way ANOVA revealed a difference between groups for L (F (24,370) = 8.92, *p* < 0.001, ŋ_p_^2^ = 0.083), L_M-L_ (F (24,370) = 7.27, *p* < 0.001, ŋ_p_^2^ = 0.069), and L_A-P_ (F (24,372) = 7.74, *p* < 0.001, ŋ_p_^2^ = 0.082). There was a significant interaction between group and task for L (*p* = 0.035, ŋ_p_^2^ = 0.034) and L_M-L_ (*p* = 0.03, ŋ_p_^2^ = 0.035), but not for L_A-P_, *p* > 0.05. (Figure 2). The post hoc showed that, during the ST, the subjects from the advanced-PD group showed significantly higher values of all parameters compared to the early PD and control groups (*p* < 0.001); this is shown in Figure 2 and Figure 3, In the advanced-PD group, DT significantly reduced L_M-L_ (*p* = 0.027), whereas the control group showed a significant increase in L (*p* = 0.039) and L_A-P_ (*p* = 0.013) compared to the single task. In the early PD group, we did not find a significant difference between single and dual tasks, although L, L_A-P_, and L_M-L_, tended to be higher in the DT test. No significant differences between ST and DT were observed in the case of the advanced-PD subjects for L and L_A-P_, and in controls for L_M-L_ (*p* > 0.05). During the DT, subjects from the advanced-PD group showed higher L, L_M-L_, and L_A_-_P_ values compared to the early PD and control groups (Figure 3D), but without statistical significance (*p* > 0.05).

### 3.4. The Relationship between COP Parameters and the Disease Stage, Cognitive Function, and LEDD

**Figure 4:**
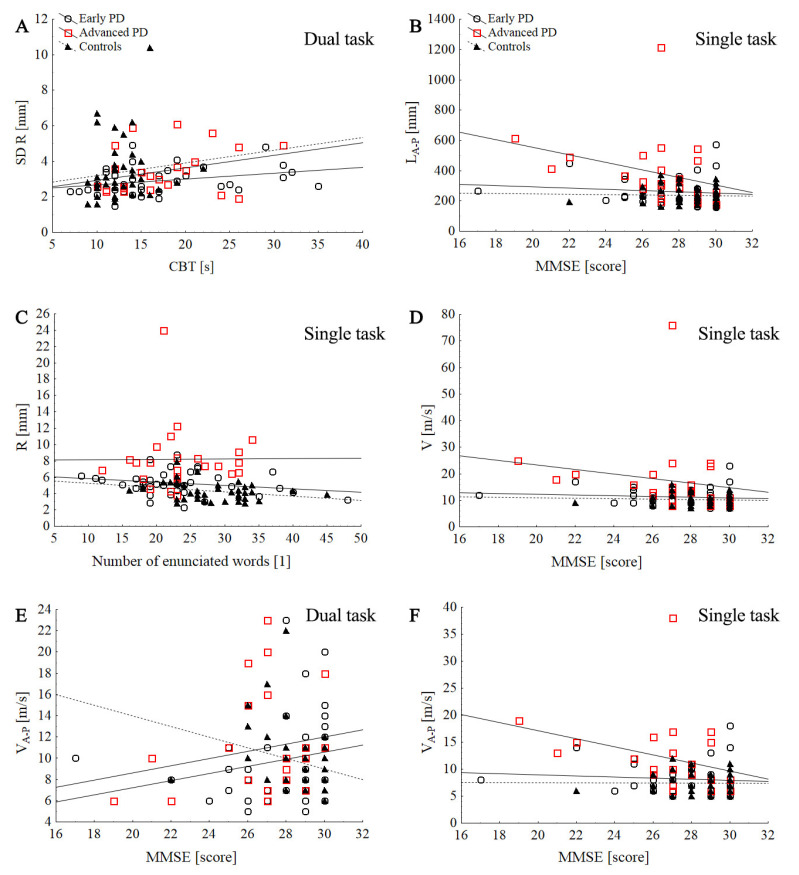
Scatter plot of the association between the stabilographic parameters with counting-backward test (CBT) and MMSE score in control and PD groups during single and dual tasks: SD R dual task (**A**), L_A-P_ single task (**B**), R single task (**C**), V single task (**D**), V_A-P_ dual task (**E**), V_A-P_ single task (**F**).

No significant correlations were observed between the H–Y stage and cognitive function (MMSE, CBT, and number of words enunciated in the dual-task condition). However, H–Y stage positively correlated with COP parameters: R (R = 0.52; *p* < 0.000), SD R (R = 0.46; *p* < 0.000), P (R = 0.51; *p* < 0.000), L (R = 0.41; *p* = 0.001), V (R = 0.41; *p* = 0.001), SD V (R = 0.39; *p* = 0.003), L_M_-_L_ (R = 0.43; *p* = 0.001), V_M-L_ (R = 0.40; *p* = 0.001), SD V_M-L_ (*p* = 0.003), V_A-P_ (R = 0.40; *p* = 0.001), and V_A-P_ (R = 0.26; *p* = 0.002) during ST. In the early PD group, the CBT was positively correlated with the SD R during DT (R = 0.34; *p* = 0.036). No significant correlations were observed between the COP parameters and the MMSE, number of words enunciated in the dual-task condition. LEDD was positively correlated with MMSE test (*p* = 0.015). In the advanced-PD group, MMSE was negatively correlated with stabilographic parameters during DT: L (R = −0.44, *p* = 0.031), V (R = −0.44; *p* = 0.03), SD V (*p* = 0.045), L_A-P_ (R = −0.5; *p* = 0.011), V_A-P_ (*p* = 0.015), and SD L_A-P_ (*p* = 0.018). Furthermore, LEDD was positively correlated with stabilographic parameters in ST:P (R = 0.45; *p* = 0.028), L_M-L_ (R = 0.46; *p* = 0.022), V_M-L_ (R = 0.46; *p* = 0.025), and SD V_M-L_ (R = 0.45; *p* = 0.029). In the control group, no significant correlation was observed between the MMSE, number of words enunciated in the dual-task condition, and stabilographic parameters in ST and DT (*p* > 0.05). Furthermore, CBT was correlated with L_M-L_ (*p* = 0.041), V_M-L_ (*p* = 0.042), and SD V_M-L_ (*p* = 0.038) during DT (Figure 4).

The ANCOVA results showed a significant effect of age on V_A-P_ (*p* = 0.019), SD V_A-P_ (*p* = 0.034), and L_A-P_ (*p* = 0.022) and a significant effect on group for whole stabilographic parameters during ST conditions: R (*p* < 0.001), SD R (*p* < 0.001), P (*p* = 0.009), V (*p* < 0.001), SD V (*p* = 0.002) V_M-L_ (*p* = 0.006) V_A-P_ (*p* < 0.001), SD V_M-L_ (*p* = 0.01), SD V_A-P_ (*p* < 0.001), L (*p* < 0.001), L_M-L_ (*p* = 0.004), and L_A-P_ (*p* < 0.001). During DT, we found a significant effect of age on R (*p* = 0.004), P (*p* = 0.012), V (*p* = 0.02), SD V (*p* = 0.036), V_M-L_ (*p* = 0.04), V_A-P_ (*p* = 0.037), SD V_A-P_ (*p* = 0.019), and L_A-P_ (*p* = 0.03), but not for group (*p* > 0.05; see (Table 2)).

### 3.5. Relationship between Age and COP Parameters, Cognitive Function

**Figure 5:**
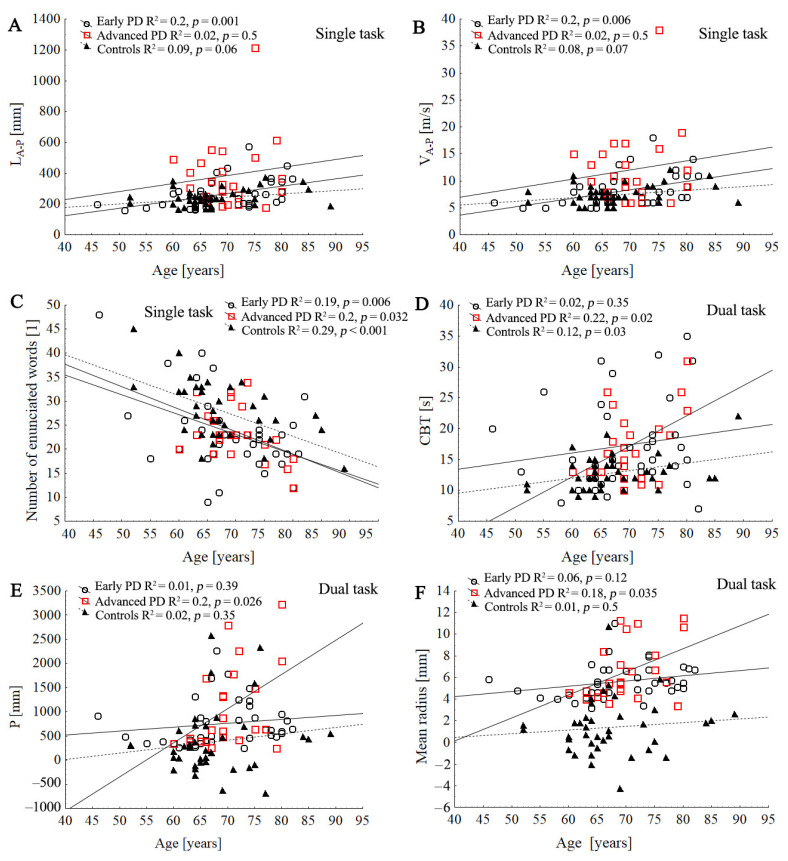
Comparison of three regression slopes by means of an ANCOVA. Scatter plot of the association between the age with stabilographic parameters and cognitive function tests in control and PD groups during single and dual task: L_A-P_ (**A**), V_A-P_, (**B**), number of enunciated words (**C**), CBT (**D**), P (**E**), mean radius (**F**). Lines represent the slope of the linear regression between the subjects’ age and the dependent variable.

Age was negatively correlated with number of words enunciated in the dual-task condition in all groups; however, it was positively correlated with CBT in the advanced-PD and control groups (Figure 5). In the early PD group, age was negatively correlated with MMSE test (R = 0.34; *p* = 0.04) and positively correlated with stabilographic parameters: P (R = 0.30; *p* = 0.047), L (R = 0.40; *p* = 0.013), V (R = 0.42; *p* = 0.09), SD V (R = 0.33; *p* = 0.046), L_A-P_ (R = 0.44, *p* = 0.006), V_A-P_ (R = 0.44; *p* = 0.006), and SD V_A-P_ (R = 0.40; *p* = 0.012) during ST condition.

In the advanced-PD group, age was positively correlated with stabilographic parameters only during dual conditions (Figure 5). In the control group, age was significantly associated with SD R (R = 0.34; *p* = 0.033), L (R = 0.32; *p* = 0.046), SD V (R = 0.34, *p* = 0.033), and SD V_A-P_ (R = 0.32; *p* = 0.045) during ST, and it was associated with L (R = 0.33; *p* = 0.035), V (R = 0.34; *p* = 0.033), and L_A-P_ (R = 0.32; *p* = 0.04) during DT conditions.

## 4. Discussion

Our study investigated the influence of performing an additional cognitive task on COP displacement in early and advanced stages of patients with PD compared to age-matched healthy elderly.

As previously described, postural instability in PD is associated with progressive loss of dopamine signaling which may result in abnormal peripheral sensory and motor integration related to basal ganglia dysfunctions [37,38,39,40,41]. As we expected, a higher stage of PD was associated with a greater level of postural instability in quiet stance [40,41]. Along these lines, we found that the advanced-PD group had significantly higher values of all stability parameters in both AP and ML directions, as compared to the early PD group and healthy controls during the eyes-open task.

In contrast to our hypothesis, we did not find significant intergroup differences in dual-task conditions for the stabilographic parameters. We also found that, in the advanced-PD group, dual task reduced postural sway, whereas the control group, under the same conditions, showed a significant increase in postural sway compared to the eyes-open condition, *p* < 0.05. In the early PD group, we did not find a significant differences between ST and DT conditions. However, a significant interaction was observed between group and task for COP parameters, except for the area of stabilogram and anterior–posterior length and sway velocity variables. The effect sizes were medium to large, suggesting that dual-task interference might be taken as evidence for interference at the level of information processing and decision-making performance [42].

It has been well documented that, when two tasks are performed simultaneously, there is competition for central processing attentional resources, resulting in performance deterioration of one or both tasks [15,17]. Our results are consistent with the study of Holmes et al., who showed that PD patients may over-constrain their posture in order to focus attention on the cognitive tasks without losing their balance [15]. Previous studies indicate that, in advanced stages of the disease, compensation is less likely to occur than in earlier stages of the disease and, if present, likely takes place outside the basal ganglia [43,44]. Namely, reduced body sway among those with advanced PD may be explained by the fact that cortical resources are directed to the cognitive tasks and patients with PD may stabilize their posture beyond normal levels to prevent threats to balance [15]. Furthermore, patients with advanced stages of PD may display postural freezing episodes, which occur where motor or cognitive (attention and anxiety) information needs to be processed [45,46]. Reduced postural sway during dual task is also probably the result of a combination of factors, including increased musculoskeletal stiffness, as well as higher co-contractions of antagonistic muscle groups [47,48].

We hypothesized that reduced postural in ML direction in the advanced-PD group may reflect the activation of a compensatory mechanism for maintaining stabilizing movements during DT in order to compensate for the greater postural sway in the anterior–posterior direction during the eyes-open condition. This might be a protective response to avoid forward or backward falls [49]. Conversely, Marchese et al. and Ferrazolli et al. reported increased postural sway during dual-task performance (COP area and SD of the COP, respectively), whereas no differences in the COP path in the anterior–posterior and medio-lateral directions were observed [21,50]. Another study in 25 PD patients and 20 controls reported that all participants shortened the mean sway radius in DT conditions compared with ST; however, only healthy subjects presented less transversal COP sway in dual-task conditions than in single-task conditions [6].

These discrepancies may be explained by the type of secondary task, complexity of the dual task, and disease severity (2,5-3 H–Y). It is worth noting that data for patients with PD in early and moderate to severe stages were not reported separately and neither of studies included patients with PD in stage 4 of H–Y.

Furthermore, in the early PD group, a higher LEDD was associated with greater postural sway in anterior–posterior direction, whereas in the advanced-PD group, in medio-lateral directions, it was associated with both ST and DT conditions. Our results supported the hypothesis that the length of the stabilogram and the sway velocity could be sensitive indicators of balance impairment and fall risk in PD [51,52]. Previous studies suggest that the administration of levodopa seemed to destabilize the patients with PD, especially in regard to the lower-back region [53]. Revilla et al. reported that dopaminergic medication reduced postural sway for patients with advanced PD with lower fall risk, whereas it had detrimental effects on postural sway for those with a higher fall risk [54].

The data in the present study showed that, in the early PD group, dual tasking increased stabilographic parameters, but without statistical significance. This may be explained by the fact that patients with early PD did not have to recruit significantly more attentional strategies to maintain the postural stability [23]. Subjects in the early stage of PD compensate for basal ganglia dysfunction with greater functional connectivity between the subthalamic nucleus and supplementary motor loop; this is not observed in individuals with freezing of gait who are mostly on advanced stages [54].

In contrast, in the control group, dual tasking significantly increased postural sway and prioritized the cognitive task, and such results are consistent with previous studies [8]. Focusing intentionally on postural control during two tasks performed simultaneously may also be associated with less automatic control of balance and decline in postural control [55]. This assumption is consistent with the capacity-sharing model that proposes that, as attention is divided, the performance of two attention-demanding tasks may be altered even if capacity limits are not exceeded [56].

No significant differences in any stabilographic parameters were observed between the controls and early PD group in all two conditions. Similarly, Fernandes et al. found no differences in medio-lateral and anterior–posterior velocity in the CoP displacement between the ST and DT conditions [23]. However, Chen et al. reported an increase of root-mean-square values of sway acceleration during cognitive task performance, but without significant differences in ST between the control group and the early untreated-PD group (H–Y 1.7) [57].

In our study, early and advanced patients with PD showed significantly worse cognitive performance compared to the controls, and this may suggest a subtle delay in cognitive functioning associated with executive function [58]. Interestingly, our study also found that, although subjects with advanced PD had higher stages on the H–Y, no significant intergroup differences were observed for all cognitive tests.

This may be explained by the fact that impairment in executive function and attention abilities, as the most prominent cognitive changes in PD, may occur even in the early stages of PD [59,60]. Our results are consistent with those of the previous studies, which suggest no significant relationship between executive deficits and the H–Y score in patients with PD [61]. Conversely, Ridder et al. have suggested significant cognitive deficits (especially executive dysfunction) in those with more advanced disease according to Hoehn–Yahr [62]. Some studies suggest that PD-related executive dysfunction is not directly correlated with motor dysfunction [63], although it has been linked with gait disturbance [64].

In the advanced-PD group, a lower MMSE score was associated with greater postural sway in the anterior–posterior direction during ST, which may indicate that some part of attention is always required in an upright stance [7]. Significant correlations between COP parameters and executive function were observed only in the control and early PD groups (SD R); this may suggest shared neural pathways. The lack of significant correlations between CBT, number of enunciated words during dual task, and stabilographic parameters in the advanced-PD group may suggest that the association between executive function and postural control could be more prominent when cognitive tasks become more complex [65]. Moreover, the complexity of the cognitive task in dual tasking can significantly influence the performance of motor and cognitive tasks in subjects with advanced PD [16].

As previously described, age-related deficits in the postural control and cognitive system may accelerate or accompany motor and executive declines in patients with PD [12]. Advanced age was related to greater postural sway and lower performance of executive functions in all groups. Our results showed significant effects of age and group on stabilographic parameters in the anterior–posterior direction during ST (length of stabilogram and sway velocity in anterior–posterior directions), and this may confirm greater postural sway in the AP direction than ML over time [28]. Previous studies suggest that a reduction in AP postural dynamics may result from greater instability in the AP direction, associated with decreased knee flexion and greater difficulty initiating ankle dorsiflexion to maintain balance [28]. In the early PD and control groups, age was significantly associated with greater postural sway, especially in the AP direction during ST, whereas in the advanced-PD group with stabilographic parameters, it was significant only during dual condition. Namely, elderly subjects can prioritize postural stability in the AP direction to recover balance [66], but older adults with a history of falls were more likely to make use of two or more postural-adjustment strategies to execute the task [12].

Our study did not evaluate the impact of gender difference in postural control; however, the consequences of aging in relation to postural stability may differ between men and women. Some studies indicate that both healthy elderly women and women with PD are more susceptible to falls [67].

Understanding the impact of postural-control strategies during DT training in PD has broad clinical implications. A rehabilitation program with dual tasks may improve motor learning and neuroplasticity at the level of synaptic connections and neural circuits, potentially being a key point in a therapeutic approach to PD patients. A large number of repetitive and targeted exercises promote brain remodeling and improve the automation level of actions [4,68]. An experimental animal study conducted by Binda et al. showed that exercise can induce limited endogenous nerve-repair mechanisms, increase axons and dendritic branches, speed up information processing, and improve performance [69]. Similarly, a systematic review conducted by Li confirmed that dual-task training was effective in improving gait performance, motor symptoms, and balance in patients with PD relative to other forms of training or non-intervention [70].

PD patients with low-level balance confidence during DT may over-constrain their posture through an increase of postural stiffness in order to release attentional resources for cognitive performance during an additional task [6]. These assumptions confirm our findings, especially in relation to the advanced-PD group. Thus, the choice of compensation strategies for balance and gait impairment in PD should be tailored to the individual patient in terms of the clinical context in which the strategy needs to be applied [71].

Regarding the application of our results to rehabilitation, clinicians need to take into account the disease stage, hypokinesia/stiffness level, cognitive status, patients’ demographics, and treatment with antiparkinsonian drugs; they also need to consider testing more than one-task activity in various conditions.

There are some limitations in our study. First, the cognitive tasks that were chosen might not have been complex enough to detect differences between the early PD and control group. It is assumed that a low level of cognitive-task difficulty is sufficient to shift attention away from the postural domain and improve postural stability without causing resource competition [65], whereas higher levels of cognitive-task difficulty have opposite effect. Second, we did not assess postural sway in the eyes-closed and foam conditions, and some authors have pointed out its relevance to the sensory-induced balance change [41,72,73].

Our study was performed under static conditions; however, postural-control assessments should include both static and dynamic conditions in older adults [74]. Third, we chose only mobile patients with advanced PD to ensure that most PD patients could perform the dual task properly. In a dual task, factors such as fatigue and the complexity of the task cause the inhibition and facilitation of brain functions [75].

Another limitation is the difference in time concerning the baseline trial for the cognitive task of counting backward (20 s) vs. the dual-task conditions (50 s). Moreover, stratification by the H–Y stage was limited by the low number of patients with advanced PD, and this could influence the strength of our observations.

## 5. Conclusions

Our studies indicate that both early and advanced patients with PD showed significant delay in cognitive performance associated with executive function compared to the healthy controls. Furthermore, during additional cognitive tasks, patients with advanced stages of PD may reduce stabilographic parameters in the medio-lateral direction, and this is probably an adaptive strategy to restore balance. Age is an important factor influencing COP displacement, especially in the anterior—posterior direction. Our results indicate the need to have different stages of PD and accompanying age-related changes taken into account when developing effective cognitive and motor trainings for improving functional mobility, particularly in the later stage of disease. Future research should identify differences in dual-task prioritization between the four H–Y stages of patients with PD and continue to investigate muscle leg activity during perturbed standing balance in medio-lateral and anterior–posterior directions.

## Figures and Tables

**Table 1:** Subjects’ characteristics.

	Patients with PD	Controls	*p*	Early PD	Advanced PD	*p*
Number of subjects (*n*)	62	40	-	38	24	-
Age (years)	69.46 ± 7.5	67.2 ± 7.7	0.7	69.0 ± 8.6	70.1 ± 5.4	0.6
Gender (male/female)	36/26	15/25	0.07	22/16	14/10	0.8
Mini Mental State Examination	27.15 ± 2.7	28.5 ± 1.7	0.003	27.4 ± 2.7	26.8 ± 2.7	0.34
Counting-backward test	17.25 ± 6.6	12.9 ± 2.7	<0.001	17.2 ± 7.3	17.2 ± 5.6	0.94
Number of enunciated words in dual task	23.54 ± 6.6	28.1 ± 6.0	<0.001	23.5 ± 8.1	23.6 ± 5.7	0.97
Disease duration (years)	5.61 ± 4.8	-	-	4.0 ± 2.9	8.17 ± 6.0	<0.001
Levodopa equivalent daily dose (mg)	513.24 ± 442.2	-	-	373.9 ± 259.7	699.1 ± 560.0	0.016
Hoehn–Yahr stage (H–Y) (0–4)	2.3 ± 0.9	-	-	1.7 ± 0.5	3.25 ± 0.4	<0.001
Stage 1 (*n*)	12	-	-	-	-	-
Stage 2	25	-	-	-	-	-
Stage 3	18	-	-	-	-	-
Stage 4	6	-	-	-	-	-
Rehabilitation treatment						
Yes	29	-		16	13	
No	32	-		21	11	

**Table 2:** Effect of age on stabilographic parameters during single and dual task.

	Single Task		Dual Task	
	Age	Group	Age	Group
R [mm]	F (1.97) = 1.9	F (2.97) = 22.5 ***	F (1.97) = 8.6 **	F (2.97) = 1.2
SD R	F (1.97) = 2.5	F (2.97) = 15.6 ***	F (1.97) = 2.1	F (2.97) = 1.8
P [mm^2^]	F (1.97) = 0.1	F (1.97) = 5.0 **	F (1.97) = 6.5 *	F (2.97) = 1.7
V [m/s]	F (1.97) = 2.9	F (2.97) = 8.0 ***	F (1.97) = 5.6 *	F (2.97) = 1.0
SD V	F (1.97) = 2.3	F (2.97) = 6.8 **	F (1.97) = 4.5 *	F (2.97) = 1.1
V_M-L_ [m/s]	F (1.97) = 1.3	F (2.97) = 5.4 **	F (1.97) = 4.1 *	F (2.97) = 1.5
V_A-P_ [m/s]	F (1.97) = 5.7 *	F (2.97) = 10.3 ***	F (1.97) = 4.5 *	F (2.97) = 0.6
SD V_M-L_	F (1.97) = 0.1	F (2.97) = 4.8 *	F (1.97) = 2.8	F (2.97) = 1.9
SD V_A-P_	F (1.97) = 4.6 *	F (2.97) = 10.0 ***	F (1.97) = 5.7 *	F (2.97) = 0.7
L [mm]	F (1.97) = 2.9	F (2.97) = 7.9 ***	F (1.97) = 5.4	F (2.97) = 1.0
L_A-P_ [mm]	F (1.97) = 5.4 *	F (2.97) = 9.9 ***	F (1.97) = 4.9 *	F (2.97) = 0.6
L_M-L_ [mm]	F (1.97) = 1.2	F (2.97) = 5.8 **	F (1.97) = 4.7	F (2.97) = 1.5

* Indicates *p* < 0.05; ** indicates *p* < 0.01; *** indicates *p* < 0.001.

## Data Availability

All the data are presented within the manuscript.
